# Population correlates of circulating mercury levels in Korean adults: the Korea National Health and Nutrition Examination Survey IV

**DOI:** 10.1186/1471-2458-14-527

**Published:** 2014-05-29

**Authors:** Seongbeom Cho, David R Jacobs, Kyong Park

**Affiliations:** 1 College of Veterinary Medicine and Research Institute for Veterinary Science, Seoul National University, Seoul, Republic of Korea; 2 Division of Epidemiology and Community Health, School of Public Health, University of Minnesota, Minneapolis, MN, USA; 3 Department of Food and Nutrition, Yeungnam University, Gyeongsan, Gyeongbuk, Republic of Korea

**Keywords:** Blood mercury, Correlates, Fish intake, Korean adults

## Abstract

**Background:**

Prior studies focused on bioaccumulation of mercury (Hg) and on large, long-lived fish species as the major environmental source of Hg, but little is known about consumption of small-sized fish or about non-dietary determinants of circulating Hg levels. The purpose of this study was to evaluate whole blood mercury concentration (WBHg) and its major dietary and non-dietary correlates in Korean adults.

**Methods:**

We analyzed cross-sectional data from 3,972 (male = 1,994; female = 1,978) participants who completed the Korean National Health and Nutrition Examination Survey IV, 2008 to 2009. Relevant factors included diet, geographic location of residence, demographics, and lifestyle. WBHg concentration was measured using cold-vapor atomic absorption spectrometry. Multivariable linear models assessed independent correlates of dietary and non-dietary factors for WBHg levels.

**Results:**

Median levels of WBHg were 5.1 μg/L in men and 3.7 μg/L in women. Higher levels of fish/shellfish intake were associated with higher levels of WBHg. Higher consumption of small-sized fish was linked to higher levels of WBHg. Non-dietary predictors of higher WBHg were being male, greater alcohol consumption, higher income and education, overweight/obesity, increasing age, and living in the southeast region.

**Conclusions:**

Both dietary and non-dietary factors were associated with WBHg levels in the Korean population. There is significant geographic variation in WBHg levels; residents living in the mid-south have higher WBHg levels. We speculate that uncontrolled geographic characteristics, such as local soil/water content and specific dietary habits are involved.

## Background

Fish consumption is relatively high and has been increasing steadily in the Asian population [1-3]. Fish intake is a major source of mercury (Hg) exposure in humans. Hg is a heavy metal that is released into the air from coal-fired power plants and other industrial processes, and bioaccumulates in the marine food chain, binding to tissue proteins depending on body size, lifespan, or levels of environmental contamination.

The potential independent correlates of Hg have not been well-characterized in Korea. There may be differences in human Hg concentrations depending on socio-economic level [4], residential area [4-6], traditional culture or ethnicity [4,7-9], lifestyle characteristics [10], and the types of fish and seafood typically consumed [6,9]. Prior Korean Hg studies have generally focused on the acute toxic effects of Hg [11-13] and have been conducted in geographically limited areas [14-16]. Others have simply presented the average levels in subjects without considering the types of seafood or geographic variations [17-19].

Understanding the independent dietary and non-dietary determinants of Hg status is important for elucidating the biologic mechanisms related to chronic exposure to Hg and for identifying potential interventions that might improve the health benefits of fish consumption. The present study examined both dietary and non-dietary correlates of Hg status in a cross-sectional sample of 3,397 Korean men and women aged 20–87 years who participated in the Korea National Health and Nutrition Examination Survey (KNHANES IV). Generally, Korean adults frequently consume fish in greater quantities than do Western populations [1-3]. Thus, we identified the types of fish most frequently consumed and associated with higher levels of whole blood mercury concentration (WBHg), and investigated the independent demographic, lifestyle, and geographic determinants of WBHg levels.

Methyl mercury, mostly from fish intake, may accumulate over time in human muscle tissue, so is expected to be positively correlated with age. Region-specific dietary habits may also be related to Hg levels, such as serving whale/shark meat in dishes for a traditional ceremony or locally caught types of fish. In addition, there is now widespread concern over red meat consumption among middle aged Korean population, who tend to reject red meat in favor of other alternatives such as fish, particularly sashimi for alcohol dishes. Considering these characteristics, we hypothesized that Hg levels are closely associated with higher intakes of small marine fish as well as large predator species in the population with high and varied fish consumption, and some geographic variation can be observed, controlling diet.

## Methods

### Population and design

We analyzed data from the KNHANES IV (2008–2009), a nationwide cross-sectional survey, using a complex, stratified, multistage, probability-cluster sampling of the non-institutionalized civilian population in South Korea. Details of this survey can be found elsewhere [20,21]. Briefly, the survey collected extensive information on health and nutritional status through health interviews, health examinations, and dietary interviews administered by trained personnel. For the two years covered by the survey, there were 20,277 respondents; 9,744 in 2008 and 10,533 in 2009. KNHANES randomly selected 20% of participants at each examination to evaluate their whole blood levels of heavy metals. In this analysis, we included all participants with WBHg whose ages ranged from 20 to 87 years (n = 3,996). Among them, we excluded 1 individual with WBHg concentration >150 μg/L that could reflect exogenous contamination and 23 pregnant women. All participants provided written consent and the study was approved by the Korea Centers for Disease Control and Prevention Institutional Review Board.

### Assessment of WBHg levels

Before blood was drawn, participants were asked to fast overnight. Venous blood samples were drawn into standard commercial evacuated tubes containing sodium heparin (vacutainer). Whole blood Hg measurements were performed by a gold-amalgam collection method with DMA-80 (Milestone, Bergamo, Italy); measurements took place at the Neodin Medical Institute in South Korea. Laboratory personnel were blinded to all participant characteristics and intra-assay variability was excellent with CV = 1.59–4.86%.

### Assessment of dietary, lifestyle and geographic information

Dietary information was estimated using a food frequency questionnaire (FFQ) and a 24-hour dietary recall test. The KNHANES used a 63-item FFQ, non-quantitative in the sense that portion size information was not queried. We therefore quantified levels of fish and shellfish consumption in two separate ways: (1) by frequency according to the FFQ and (2) by recording daily intake levels from a 24-hour recall. The FFQ listed nine items regarding the frequency of fish consumption and shellfish most frequently consumed by South Koreans: mackerel, tuna, yellow fish, pollock, anchovy, seafood paste, squid, clam, and pickled seafood. Items on the FFQ were rated on a Likert-type scale ranging from “rarely” to “3 times a day”, and the frequency per food item was converted into times per week using median values. In order to obtain daily intake levels of fish and shellfish, we identified the marine species consumed by at least 1% of all participants (n = 3972) in the present sample and then calculated the individual intake levels for each item using 24-hour recall data. Consumption of tuna was estimated separately by the product preparation method (for example, canned and fresh/sashimi) because levels of Hg differ substantially according to the type of species used [21,22].

Information on demographic and lifestyle factors including age, sex, income, education, smoking, alcohol consumption, and physical activity was obtained through a health interview questionnaire. Education level was categorized as middle school or less, high school, and college or more. Smoking status was classified as never, former, or current smoker. Alcohol consumption was quantified as servings per week. Participants were asked how much time they had spent in vigorous and moderate activities and walking during the past week; activities were then weighted by frequency and duration in order to obtain the metabolic equivalents (METs)-h/week [23]. A trained technician took anthropometric measurements, including waist circumference, height, and weight. Body mass index (BMI) was calculated as weight (kg) divided by height (m^2^) squared. “Overweight/obesity” was defined based on 2000 statement from the World Health Organization for Asian population: the BMI cutoff value for overweight was 23 kg/m^2^ and for obesity was 25 kg/m^2^[24]. Waist circumference (cm) was measured as the abdominal girth midway between the iliac crest and the bottom of the rib cage, with the arms relaxed at the sides. Participants were asked to report the frequency of eating out, ranging from “rarely” to “2 times a day” in 5 categories, and ratings were converted into a “times per week” measure. Residential areas were evaluated separately and then grouped into 5 geographic regions based on similarities of both WBHg levels and geographic location: north (Seoul, Incheon, Gyeonggi-do, Gangwon-do), midwest (Daejeon, Chungcheongbuk-do, Chungcheongnam-do), mideast (Daegu, Gyeongsangbuk-do), southwest (Gwangju, Jeollabuk-do, Jeollanam-do), and southeast and island (Busan, Ulsan, Gyeongsangnam-do, Jeju island).

### Statistical analysis

A multivariable linear regression was used to examine associations of fish intake, demographic factors, and lifestyle factors, with log-transformed WBHg level as the dependent variable. For categorical variables, the Chi-square statistic was computed to compare participant characteristics with quartiles of WBHg. Population means for fish and shellfish daily consumptions were estimated and compared with quartiles of WBHg using generalized linear regression analyses. Region-specific WBHg levels were estimated and adjusted for age, sex, BMI, physical activity, smoking status, alcohol intake, household income, fish intake level (g/day), and frequency of eating out. Effect modification of the associations was tested by cross-product terms, and no significant interaction was found. Because the study analyzed data collected using a stratified, multistage probability sampling design (the KNHANES), all analyses were performed using survey procedures in SAS® (version 9.2). For all tests, the critical value for p was set at α = 0.05, two-tailed.

## Results

Table 1 gives demographic characteristics of the study participants sorted by sex (1,994 men, 1,978 women). The median concentration of WBHg was 5.1 μg/L in men and 3.7 μg/L in women. Distributions of age, income level, and residential area did not differ by sex. The prevalence of overweight/obesity and smoking was significantly greater in men than in women. In addition, men were more likely to frequently consume alcohol and eat out and to have higher levels of physical activity (MET-h/week) and education compared to women.

**Table 1:** Demographic characteristics of participants from the KNHANES (2008–2009)

	**N**	**Sex**				**p-value**
		**Men (n = 1994)**		**Women (n = 1978)**		
Range of mercury (μg/L)	3972	0.90-56.97		0.88-33.93		-
Mean mercury (μg/L)	3972	6.32	± 0.12	4.25	± 0.07	<0.001
Age (years, %)						0.9
20-29	775	396	(20)	379	(19)	
30-39	795	404	(20)	391	(20)	
40-49	800	400	(20)	400	(20)	
50-59	790	391	(20)	399	(20)	
≥60	812	403	(20)	409	(21)	
Overweight/obesity (BMI ≥ 23, %)	1242	691	(35)	551	(28)	<0.001
Smoking status (%)						<0.001
Never	2126	395	(20)	1731	(87)	
Former	807	695	(35)	112	(6)	
Current	1033	899	(45)	134	(7)	
Alcohol intake (n/week)	3964	1.33	± 0.03	0.53	± 0.02	<0.001
MET-h/week						<0.001
<20	1693	747	(38)	946	(48)	
20-39	763	389	(20)	374	(19)	
≥40	1493	846	(42)	647	(33)	
Eating-out (n/week)	3450	5.26	± 0.13	2.90	± 0.10	<0.001
Income level (%)						0.1
Low	1004	498	(26)	506	(26)	
Mid low	968	481	(25)	487	(25)	
Mid high	965	461	(24)	504	(26)	
High	964	512	(26)	452	(23)	
Education (%)						<0.001
Middle school or less	1340	556	(28)	784	(40)	
High school	1490	782	(40)	708	(36)	
College or more	1125	642	(32)	483	(24)	
Residence area (%)						0.9
Midwest	475	234	(12)	241	(12)	
North	1873	944	(47)	929	(47)	
Southwest	504	248	(13)	256	(13)	
Mideast	422	207	(10)	215	(11)	
Southeast and an island	698	361	(18)	337	(17)	

Values are n (%) or mean ± SE, except the range of mercury.

Table 2 summarizes Hg levels by quartile and according to demographic characteristics. The highest levels of WBHg were recorded in individuals in the 40–59-year-old age group. Higher levels of WBHg were associated with overweight/obesity, current smoking, frequent alcohol drinking and eating out, higher levels of income and education, and residence in the mideast or southeast/island areas of South Korea.

**Table 2:** Demographic characteristics of participants from the KNHANES (2008–2009), according to quartile of blood mercury levels (n = 3972)

	**Geometric mean of mercury**	**N**	**Quartile of blood mercury level**				
			**Q1 (0.88-3.06)**	**Q2 (3.07-4.36)**	**Q3 (4.37-6.41)**	**Q4 (6.42-56.97)**	**p-value**
	**(μg/L)**		**%**	**%**	**%**	**%**	
Age (years)							<0.001
20-29	3.81	775	26	21	19	11	
30-39	4.48	795	18	23	21	19	
40-49	5.04	800	15	19	20	27	
50-59	5.15	790	14	18	22	25	
≥60	4.15	812	27	19	18	18	
Men	5.35	1994	32	41	57	72	<0.001
Overweight/obesity (BMI ≥ 23)	5.13	1242	25	27	32	42	<0.001
Smoking status							<0.001
Never	3.99	2126	68	61	49	37	
Former	5.22	807	14	17	21	29	
Current	5.14	1033	18	22	30	34	
Alcohol intake (≥1/week)	5.68	938	13	17	26	38	<0.001
Eating-out (≥1/day)	4.88	713	17	21	24	27	<0.001
MET-h/week							0.003
<20	4.29	1693	48	43	42	39	
20-39	4.60	763	19	20	19	20	
≥40	4.69	1493	33	37	39	41	
Income level							<0.001
Low	4.14	1004	30	27	24	22	
Mid low	4.45	968	24	26	25	23	
Mid high	4.43	965	26	26	23	24	
High	5.06	964	20	21	28	31	
Education							<0.001
Middle school or less	4.36	1340	39	31	33	33	
High school	4.38	1490	37	42	38	34	
College or more	4.82	1125	24	27	29	33	
Residence area							<0.001
Midwest	3.67	475	18	12	11	7	
North	4.23	1873	51	51	48	38	
Southwest	4.48	504	13	12	13	13	
Mideast	5.36	422	8	10	10	14	
Southeast and an island	5.52	698	10	15	18	28	

Table 3 gives the most frequently consumed species of fish listed according to the levels of WBHg in the present study sample. Overall, higher intakes of white fish were associated with higher levels of WBHg. In particular, consumption of both yellow corvina (p < 0.01) and halibut (p < 0.001) were significantly associated with higher levels of WBHg and showed dose–response relationships. Intake of most oily fish types showed positive associations with levels of WBHg. Among oily fish, anchovy and mackerel were most frequently consumed and also showed significant positive associations with levels of WBHg (p = 0.01 and p < 0.01, respectively). Consumption of fresh tuna was significantly associated with higher levels of WBHg (p < 0.001) whereas consumption of canned tuna was not (p = 0.4). In Table 3, the values may have high within person variance due to single 24 hour recall, rather than multiple measurements.

**Table 3:** Daily fish and shellfish consumption levels according to blood mercury levels in a sample (n = 3972) from the KNHANES (2008–2009); data from 24-hour recall

	**Population mean**^**a**^	**Consumer mean**^**b**^	**Number of consumers**	**Quartile of blood mercury level**^**a**^				**P for trend**
				**Q1**	**Q2**	**Q3**	**Q4**	
**White fish**								
Pollock^c^	5.8	56.8	354	4.4	4.8	7.7	6.2	0.1
Yellow corvina	3.3	57.0	203	2.4	3.0	3.1	4.8	0.006
Cutlassfish	1.6	37.8	143	0.9	1.9	1.6	1.8	0.2
Halibut	1.2	83.0	49	0.6	0.8	0.8	2.5	<0.001
Cod	0.4	40.7	34	0.2	0.3	0.5	0.6	0.1
Flounder	0.5	57.2	28	0.4	0.7	0.5	0.3	0.6
Sea bream	0.7	91.5	28	0.7	0.7	0.5	1.1	0.6
Monk fish	0.5	102.4	18	0.2	0.5	0.9	0.6	0.3
Arabesque greenling	0.5	112.2	15	0.9	0.2	0.7	0.1	0.3
**Oily/fatty fish**								
Anchovy	7.3	15.1	1691	6.8	6.7	7.5	8.3	0.01
Mackerel^c^	5.5	64.1	298	4.0	5.0	5.7	7.3	0.009
Pacific saury	1.6	67.9	83	1.1	2.8	1.6	0.9	0.4
Eel	1.2	64.4	66	1.2	1.0	0.8	1.9	0.3
Tuna (fresh)	0.9	58.6	53	0.5	0.3	0.8	2.0	<0.001
Salmon	0.2	28.8	30	0.1	0.2	0.3	0.4	0.08
Spanish mackerel	0.5	80.9	22	0.1	0.5	1.2	0.3	0.7
**Other fish**								
Fish paste	5.3	36.5	506	5.1	5.7	4.9	5.6	0.8
Tuna (canned)	1.6	35.9	154	1.7	1.1	1.7	1.9	0.4
Skate	0.9	75.6	42	0.4	1.4	1.0	0.8	0.9
Rockfish	0.5	44.7	41	0.3	0.6	0.3	0.9	0.1
Filefish	0.5	48.9	39	0.1	0.3	0.7	1.1	0.003
Loach	0.8	89.6	33	0.6	0.8	1.0	1.0	0.5

^a^Mean consumption (g/day; includes that of non-consumers) of fish and seafood. Total intake level of each food item divided by total N.

^b^Mean consumption (g/day; including only consumers) of fish and seafood. Total intake level of each food item divided by consumer N.

^c^Mackerel and Pollock were independent correlates of whole blood mercury in a multivariable-adjusted analysis, including adjustments for all fish items asked in FFQ. This multivariable-adjusted analysis was conducted using FFQ data.

Table 4 provides comparisons of levels of fish and shellfish consumption for weekly (FFQ) and daily (24-hour recall) intake and according to WBHg levels. Higher levels of fish and shellfish intake were associated with higher levels of WBHg, according to both consumption measures. These associations were particularly evident in the consumption of white fish, oily fish, and shellfish (p < 0.001).

**Table 4:** Levels of fish and shellfish consumption in a participant sample (n = 3972) from the KNHANES (2008–2009) according to blood mercury levels

	**Population mean**^**a**^	**Consumer mean**^**b**^	**Number of consumers**	**Quartile of blood mercury level**^**a**^				**P for trend**
				**Q1**	**Q2**	**Q3**	**Q4**	
**Intake levels from 24-h recall (g/day)**								
Total fish^c^	41.4	58.7	2458	32.9	39.4	43.8	50.0	<0.001
White fish	14.4	65.2	770	10.7	12.9	16.3	17.9	<0.001
Oily/fatty fish	17.3	31.5	1909	13.8	16.6	18.0	21.0	<0.001
Other fish	9.7	46.1	735	8.3	9.9	9.5	11.2	0.08
Shellfish and other seafood^d^	17.9	42.8	1452	13.7	16.9	17.9	23.2	<0.001
**Intake levels from FFQ (n/week)**								
Total fish^e^	5.1	5.1	3382	4.4	5.2	5.2	5.6	<0.001
White fish (yellow corvina, pollock)	1.1	1.2	3088	0.8	1.0	1.1	1.3	<0.001
Oily/fatty fish (mackerel, anchovy)	2.9	3.0	3306	2.5	3.0	2.9	3.2	<0.001
Other fish (tuna, fish paste)	1.1	1.9	2759	1.05	1.14	1.14	1.09	0.8
Shellfish and other seafood (squid, clam, pickled seafood)	1.7	1.9	3127	1.3	1.6	1.9	2.1	<0.001

^a^Mean consumption (includes that of non-consumers) of fish and seafood. Total intake level of each food item divided by total N.

^b^Mean consumption (including only consumers) of fish and seafood. Total intake level of each food item divided by consumer N.

^c^Sum of the intake of top 22 fish types (anchovy, fish paste, pollock, mackerel, yellow corvina, tuna (canned), cutlassfish, pacific saury, eel, tuna (fresh only), halibut, skate, rockfish, filefish, cod, loach, salmon, flounder, sea bream, Spanish mackerel, monk fish, and arabesque greenling).

^d^Sum of the intake of shellfish and other seafood (clam, crawfish, crab, whelk, oyster, shrimp, conch, abalone, mussel, squid, octopus, jellyfish, sea cucumber, sea squirt, fish intestine, salted seafood).

^e^Sum of the frequency of consumption of mackerel, tuna (fresh and canned), yellow corvina, pollock, anchovy, and fish paste.

**Figure 1:**
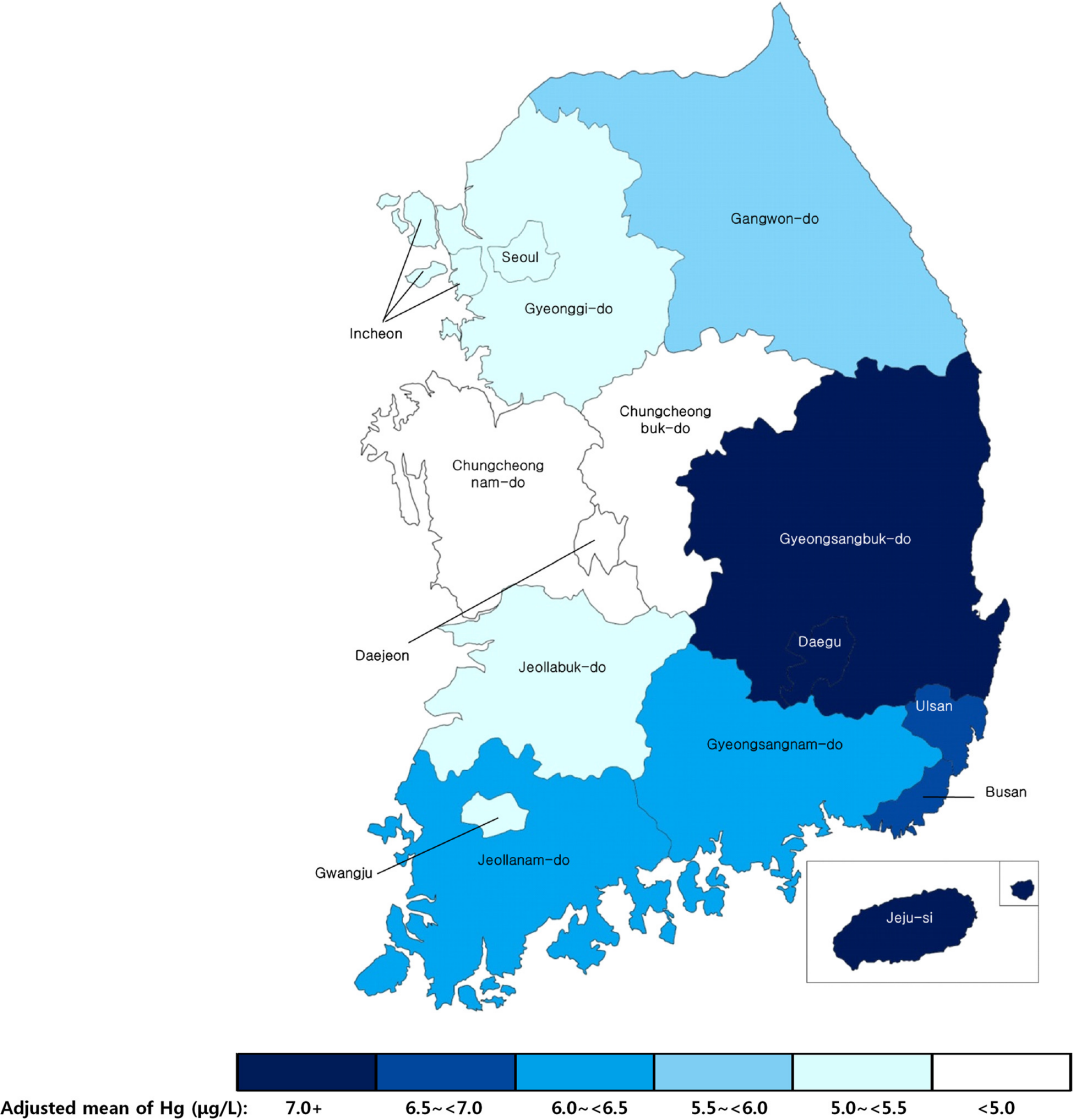
**Adjusted blood mercury levels in Korean adults.** Adjusted for age, sex, BMI, fish intake level (g/day), frequency of eating-out, physical activity, smoking status, alcohol intake, and income.

To understand whether lifestyle and environmental factors were associated with WBHg after accounting for fish and shellfish consumption, we conducted multivariable-adjusted linear regression analyses for each of the identified dietary and non-dietary factors (Table 5). Both age and gender were independent correlates of WBHg levels, even after adjusting for diet as well as other lifestyle and environmental factors. WBHg levels ranged from 11.1–12.9% higher in 40–59-year-olds than in 20–29-year-olds (p < 0.001); WBHg levels were also 9.8% higher in men than in women (p < 0.001). Several lifestyle factors were also independently associated with WBHg levels. Higher BMI was associated with higher WBHg levels, with 1.0% higher levels for each additional 1 kg/m^2^ (p < 0.001). Higher alcohol consumption was associated with higher WBHg levels with a dose–response relationship showing that, when compared to non-drinkers, levels were 9.4–9.7% higher in drinkers who had alcohol intake at least twice per week (p < 0.001). Income and education levels were also positively associated with WBHg levels, such that high-income, highly-educated participants were more likely to have higher levels of WBHg. Compared with participants in the lowest quartile of household income, those in the highest quartile had 8.1% higher WBHg levels (p < 0.001). In addition, participants with college/university education or higher had 3.7% higher WBHg levels compared to those with middle school education or less (p <0.01). Even after adjustment for each of these lifestyle factors and types of fish and shellfish consumption, we identified significant geographic variation in WBHg levels. Compared to participants living in the midwest regions of South Korea (the lowest WBHg region), those living in the southeast/island, mideast, southwest, and north, had significantly higher WBHg levels (southeast/island: 17.5% higher, p < 0.001; mideast: 16.5% higher, p < 0.001; southwest: 8.7% higher, p < 0.001; north: 5.5% higher, p < 0.001).To further examine regional differences, we calculated adjusted means of WBHg levels for each province in South Korea (Figure 1). Overall, participants in the southeast/island region appeared to have higher levels of WBHg than those in northwestern regions. The highest levels (adjusted means) of WBHg were found in Gyeongsangbuk-do (7.6 μg/L), Jeju (7.3 μg/L), Ulsan (7.1 μg/L), and Daegu (7.0 μg/L). Regions with the lowest levels (adjusted means) of WBHg were Daejeon 4.4 μg/L, Chungcheongbuk-do 4.4 μg/L, and Chungcheongnam-do 4.7 μg/L. None of these regional findings differed by age or sex.

**Table 5:** **Dietary and non-dietary correlates of whole blood mercury**^**a **^**in a participant sample (n = 3972) from the KNHANES (2008–2009)**^**b**^

	**% difference in blood mercury**	**p-value**
Age (years)		
20-29	Reference	
30-39	6.0	<0.001
40-49	11.1	<0.001
50-59	12.9	<0.001
≥60	5.6	<0.001
Men	9.8	<0.001
BMI (kg/m^2^)	1.0	<0.001
White fish intake level (g/day)		
Low tertile (0)	Reference	
Medium tertile (0 < - ≤ 40)	2.7	0.02
High tertile (>40)	3.9	<0.001
Oily/fatty fish intake level (g/day)		
Low tertile (0)	Reference	
Medium tertile (0 < - ≤ 40)	1.6	0.05
High tertile (>40)	2.6	0.03
Total shellfish intake level (g/day)		
Low tertile (0)	Reference	
Medium tertile (0 < - ≤ 20)	-1.4	0.1
High tertile (>20)	3.2	<0.001
MET-h/week		
<20	Reference	
20-39	2.6	0.01
≥40	0.8	0.3
Smoking status		
Never	Reference	
Former	0.9	0.5
Current	1.7	0.1
Alcohol intake		
Never	Reference	
≤1/mo	1.7	0.1
≤1/week	4.7	<0.001
2-3/week	9.4	<0.001
4+/week	9.7	<0.001
Income		
Low	Reference	
Mid low	3.0	0.004
Mid high	4.2	<0.001
High	8.1	<0.001
Education		
Middle school graduation or less	Reference	
High school graduation	0.4	0.7
College or more	3.7	0.003
Residence area		
Midwest	Reference	
North	5.5	<0.001
Southwest	8.7	<0.001
Mideast	16.5	<0.001
Southeast and an island	17.5	<0.001

^a^Blood mercury was logarithmically transformed as the dependent variable for the purpose of the multiple linear regression analysis. Beta-coefficient is approximately % difference in blood mercury (ln (μg/L)) from the reference category or per unit of continuous BMI).

^b^Multivariable-adjusted findings including each of the variables in the Table.

## Discussion

In this cross-sectional analysis of the general Korean population, several independent correlates of WBHg levels were identified. As expected, participants with higher intakes of fish and shellfish had significantly higher levels of WBHg and the pattern of association was a dose–response relationship. In addition, independent effects of other demographic and lifestyle factors, such as gender, age, obesity, alcohol consumption, and income and education levels were identified, accounting for the levels of fish and shellfish consumption.

Park and Lee have previously examined the association between WBHg and seafood intake levels, using 9 items from the non-quantitative FFQ of KNHANES [25]. We supplemented the non-quantitative and less detailed information from the FFQ with quantitative and detailed information from the single 24 h dietary recall, thus more fully using available KNHANES dietary information, to estimate the usual amounts of fish and seafood consumption and to add detail to the types of fish and seafood that are commonly consumed in this population. In addition, Lee et al. suggested that residents in coastal areas have relatively high levels of WBHg due to higher consumption of seafood [6]. However, our study found a significant geographic variation in WBHg levels among coastal participants even after adjusting for multiple factors.

Our study highlighted some important findings concerning Hg levels in large and small marine fish species and their relationship to Hg levels in humans. Previously, public health concern has been focused on exposure to Hg due to consumption of large predator species such as shark, swordfish, king mackerel, or tilefish [26,27]. However, in the present study, the most frequently consumed small marine species such as mackerel were associated with substantially higher WBHg levels in the Korean population. Thus, it is important to improve understanding of chronic exposure to Hg from frequently consumed small marine species. This is especially critical for populations such as Koreans, where there is high consumption of a variety of fish (of both small and large species) and shellfish. Given Korean meal patterns, correlation is expected between large and small fish intake. However, we observed higher WBHg in a multivariable-adjusted analysis, including simultaneous adjustment for all fish items asked in FFQ. Tuna, one of the most frequently consumed fish in Korea and worldwide, is known to be a major source of Hg [9,22]. Interestingly, our study showed that higher consumption of canned tuna was not associated with elevated levels of WBHg, whereas consumption of fresh tuna, such as sashimi or sushi, was associated with significantly higher levels of WBHg. The reason is unknown, but this may be related to the types of tuna since the majority of canned tuna available in the Korean market is “light-style” tuna consisting mainly of skipjack (Katsuwonus pelamis), whereas the bluefin tuna is commonly used for sashimi or sushi [28,29]. Our study results are consistent with previous research indicating that bluefin tuna has higher concentrations of Hg than does canned light tuna [22].

Several demographic and lifestyle determinants of WBHg were identified. Men had higher WBHg concentrations than women, consistent with previous findings [30]. In addition, alcohol consumption, income, and education levels were significant correlates of WBHg concentrations. The association of WBHg with alcohol is grouped here with demographic variables because many Koreans have dinner/drink meetings after work. In the face of concern about meat eating, this behavior may link alcohol and fish/sashimi consumption.

In our study, WBHg levels were positively associated with BMI after controlling for other variables. While recent study findings argue that the benefits of fish consumption may outweigh the negative effects of chronic exposure to Hg [26,31], particularly for cardiovascular health outcomes, the association between Hg exposure and overweight/obesity is not well established. Indeed, our findings contradict those of previous studies. In a study with a Brazilian sample [32], Hg exposure from fish consumption was not associated with BMI. Further, a study of the Korean elderly population residing in coastal areas showed that the waist-hip ratio was significantly correlated with blood Hg concentration, but BMI was not [19]. Thus, the mechanism of association between Hg exposure and overweight/obesity is relatively understudied and further research is warranted.

The present study found significant geographic variation in WBHg levels. South Korea is surrounded by ocean water with a long coast line on three sides. Thus, seafood is easily accessible from almost any region. Indeed, residents in a few regions, mostly located in the center of the Korean peninsula, had relatively low levels of WBHg when compared to regions located closer to the coast line. However, even after adjustment for multiple factors including fish and shellfish intake levels, significant geographic variation remained in WBHg levels. This may be due to other uncontrolled geographic characteristics, such as the type of marine species consumed, water or soil content, local ceremonial traditions or specific dietary habits (e.g., serving dishes containing whale and/or shark meat).

Our study analyzed data from the KNHANES containing data representative of random samples of the South Korean population and therefore may be generalizable to the larger population of Korean adults. In addition, we provide multivariable-adjusted estimates of WBHg, thus minimizing residual confounding while evaluating multiple potential determinants in the Korean population. Our analysis showed how each of the recorded demographic, lifestyle, geographic, and dietary factors may correlate independently with WBHg levels.

A limitation of the present study is that Hg levels were evaluated in whole blood and thus may indicate a relatively short-term exposure to Hg compared to other biomarkers such as hair or toenails. It is also possible that, although the present study evaluated multiple variables that were estimated using standardized instruments, residual confounding could still exist due to the presence of unmeasured or imperfectly measured factors. In addition, fish and seafood intake levels were evaluated using one-day 24-hour recall and the non-quantitative FFQ questionnaire, leaving open the possibility of misclassification.

## Conclusions

In summary, in addition to fish consumption, we identified several demographic and lifestyle determinants of WBHg in the Korean population. Furthermore, Koreans tend to consume large amounts of small marine species and consumption of these species was positively associated with levels of WBHg, showing dose–response type relationships. Geographic variation was also a significant determinant and our results suggest that local specific dietary habits, water, or soil may explain regional differences in WBHg levels. As expected, fish consumption was clearly the key determinant of Hg concentration, but together our results indicate that a dietary guideline to simply avoid certain types of fish may not reduce WBHg levels in the Korean population. Although evidence has emphasized that the benefit of fish consumption outweighs the risk posed by Hg exposure, these risk-benefit effects might vary, depending on the range of Hg exposure in the population. Thus, large-scale prospective studies with detailed dietary information, especially fish and seafood items, are warranted to make a definitive conclusion.

## Competing interests

The authors declare that they have no competing interests.

## Authors’ contributions

SC developed the study design, conducted the analysis, and wrote the draft of manuscript. DRJ contributed discussion, and reviewed and edited the manuscript. KP developed the study design, conducted the analysis, contributed discussion, and reviewed and edited the manuscript. All authors read and approved the final manuscript.

## Pre-publication history

The pre-publication history for this paper can be accessed here:

http://www.biomedcentral.com/1471-2458/14/527/prepub
